# Effect of Gelam Honey on the Oxidative Stress-Induced Signaling Pathways in Pancreatic Hamster Cells

**DOI:** 10.1155/2013/367312

**Published:** 2013-11-13

**Authors:** Kalaivani Batumalaie, Sher Zaman Safi, Kamaruddin Mohd Yusof, Ikram Shah Ismail, Shamala Devi Sekaran, Rajes Qvist

**Affiliations:** ^1^Department of Medicine, Faculty of Medicine, University of Malaya, 50603 Kuala Lumpur, Malaysia; ^2^Department of Molecular Biology and Genetics, Faculty of Arts and Science, Canik Basari University, Samsun, Turkey; ^3^Department of Microbiology, Faculty of Medicine, University of Malaya, 50603 Kuala Lumpur, Malaysia

## Abstract

*Background*. Oxidative stress induced by reactive oxygen and nitrogen species is critically involved in the impairment of **β**-cell function during the development of diabetes. *Methods*. HIT-T15 cells were cultured in 5% CO_2_ and then preincubated with Gelam honey extracts (20, 40, 60, and 80 *µ*g/mL) as well as quercetin (20, 40, 60, and 80 *µ*M), prior to stimulation by 20 and 50 mM of glucose. Cell lysate was collected to determine the effect of honey extracts and quercetin on the stress activated NF-**κ**B, MAPK pathways, and the Akt (ser473) activated insulin signaling pathway. *Results*. HIT-T15 cells cultured under hyperglycemic conditions demonstrated insulin resistance with a significant increase in the levels of MAPK, NF-**κ**B, and IRS-1 serine phosphorylation (ser307); however, Akt expression and insulin contents are significantly decreased. Pretreatment with quercetin and Gelam honey extract improved insulin resistance and insulin content by reducing the expression of MAPK, NF-**κ**B, and IRS-1 serine phosphorylation (ser307) and increasing the expression of Akt significantly. *Conclusion*. Gelam honey-induced differential expression of MAPK, NF-**κ**B, IRS-1 (ser307), and Akt in HIT-T15 cells shows that Gelam honey exerts protective effects against diabetes- and hyperglycemia-induced oxidative stress by improving insulin content and insulin resistance.

## 1. Introduction

Diabetes is a chronic disease that occurs when the pancreas does not produce enough insulin or when the body cannot effectively use the insulin it produces [1]. Loss of *β*-cell function caused by reduced insulin synthesis and secretion is one of the key events in the pathogenesis of type 2 diabetes. Normal *β* cells can compensate for insulin resistance by increasing insulin secretion, but insufficient compensation leads to the onset of glucose intolerance [2–4].

Chronic hyperglycemia is a cause of impaired insulin biosynthesis and secretion [5], the progression of which causes insulin resistance and is often accompanied by *β*-cell degranulation and apoptosis [6]. This process is called “glucose toxicity” and has been demonstrated in various studies in vivo [7] and in vitro [8–10]. Oxidative stress induced by reactive oxygen species (ROS) and nitrogen species produced by several biochemical pathways associated with hyperglycemia (glucose autooxidation, polyol pathway, prostanoid synthesis, and protein glycation) is critically involved in the impairment of *β*-cell function during the development of type 2 diabetes [11]. It has been reported that extracellular hyperglycemia causes intracellular hyperglycemia in *β*-cells leading to the induction of ROS [5] which becomes exacerbated, through lack of antioxidant enzymes such as catalase, glutathione peroxidase, and superoxide mutase in the pancreatic islet of diabetic animals [12].

 ROS can function as signaling molecules to activate a number of stress sensitive pathways that are linked to insulin resistance, decreased insulin secretion, and content [13], ultimately leading to late complications of diabetes [14]. This transient exposure of *β*-cells to oxidative stress interrupts the normal coupling of glucose metabolism to insulin secretion by activating stress signaling pathways [15]. The most extensively studied is the intracellular pathway leading to the activation of the nuclear factor-*κ*B (NF-*κ*B) [16], which plays a critical role in mediating immune and inflammatory responses. Following activation, NF-*κ*B translocates to the nucleus, resulting in the subsequent transcription of genes involved in the production of inflammatory cytokines that promotes the development of insulin resistance [16]. A recent study on bovine endothelial cells found that exposure to hyperglycemia initially increased the production of intracellular ROS, followed by activation of NF-*κ*B [17].

In diabetes, oxidative stress also activates the p38 mitogen activated protein kinase (MAPK) pathway which leads to activation of the serine kinases which promote the degradation of insulin receptor substrate (IRS), thus reducing the insulin signaling activity which is responsible for the development of insulin resistance [18]. A streptozotocin-induced diabetic rat model showed an increase in MAPK activity as compared to controls, which was mediated by the production of ROS [18]. Insulin receptors (IR) are cell surface receptors with *α* and *β* subunits, possessing intrinsic tyrosine kinase activity. Insulin binds to the IR and induces tyrosine autophosphorylation of the IR *β* subunit. The activated IR subsequently phosphorylate their substrates including insulin receptor substrate (IRS-1). Tyrosine phosphorylated IRS-1 recruits a number of SH2 containing signal transducers, which activate several signaling pathways. Serine phosphorylated IRS-1 (ser307) inhibits insulin signal transduction in a variety of cells by steric hindrance of the interaction between IR and IRS-1. In the presence of insulin activated serine kinases, phosphorylation of IRS-1 occurs at the serine307 site, which decreases the IRS-1 tyrosine phosphorylation, thereby decreasing the activation of insulin signaling pathway which may result in insulin resistance [19].

Recently, protein kinase B (PKB or Akt) has been shown to function in the insulin-signaling cascade by phosphorylating transcription factors which are responsible for the transcription and expression of genes related to insulin synthesis and secretion [20, 21]. Therefore Akt is necessary for normal pancreatic *β* cell function and insulin secretion. Previous studies reported that inactivation of Akt can lead to insulin resistance, decreased *β* cell mass, and impaired insulin secretion [22, 23]. Our previous study also demonstrated that the following flavonoids quercetin, chrysin, and luteolin present in the Gelam honey also had antioxidant effect [24]. The protective effect of quercetin was significantly higher than that of the other flavonoids which is consistent with the data reported by Lukačínová et al. [25]. 

The aim of our present study is to determine the effect of Gelam honey extract and quercetin on the stress activated NF-*κ*B and MAPK pathways and IRS-1 serine phosphorylation causing insulin resistance and the Akt activated insulin signaling pathway, causing increase in insulin content. 

## 2. Research Design and Methods

### 2.1. Extraction of Phenolic Compounds from Honey by Solid Phase Extraction (SPE)

Gelam honey samples (Department of Agriculture, Parit Botak, Johor, Malaysia) were subjected to base hydrolysis and extracted with ethyl acetate as described by Wahdan [26] and Seo and Morr [27]. The recovered fractions were combined and dried under nitrogen gas. 

### 2.2. Determination of the Phenolic Content

Phenolic compounds from the extract were assayed using Folin-Ciocalteau assay [28]. Briefly the extract (1 mL) was added to 10% Folin-Ciocalteu reagent (Sigma F9252) and 0.5% sodium carbonate. The contents were thoroughly mixed and allowed to stand for 2 hours. The absorbance of the blue colour that developed after 2 hours was read at 765 nm. Results were expressed in micrograms of gallic acid per gram of the extract, using a standard curve generated with gallic acid (Sigma G7384).

### 2.3. Determination of the Flavonoid Content

The total flavonoid (TF) content was determined spectrophotometrically [29]. Briefly 1 mL of honey extract or a standard solution of quercetin (Sigma Q4951) (10, 50, 100, 150, 200, 250 *μ*g/mL) in distilled water was added to a 10 mL volumetric flask containing 4 mL of double distilled water, 300 *μ*L of NaNo_2_ (5%, v/v), and 300 *μ*L of 10% AlCl_3._ The solution was allowed to stand at room temperature in the dark for 30 minutes and the absorbance was read at 430 nm. The TF content was determined using the standard curve of quercetin (*μ*g/mL). TF content was expressed as *μ*g of quercetin equivalents in 1 g of extract.

### 2.4. Cell Culture

HIT-T15 cells were cultured according to the instructions provided by ATCC (CRL-1777). The cells were cultured immediately in T-25 cm flask in the F12 K medium (ATCC 30-2004) supplemented with 10% FBS and 1% penicillin and streptomycin at 37°C (5% CO_2_ in air). Cells (3rd passage) were trypsinized and subcultured after 5 days incubation, with 80% confluency. 

### 2.5. Treatment of HIT-T15 Cells with Quercetin and Gelam Honey Extract

HIT-T15 cells (5 × 10^5^) were pretreated with Gelam honey extract (20, 40, 60, 80 *μ*g/mL) and quercetin (20, 40, 60, 80 *μ*M) for 24 hours; medium was then replaced with fresh medium. Glucose (Sigma G8769) (20 or 50 mM) was added and the cells were incubated for another 24 hours. To investigate inhibitory effects on Akt signaling pathway, cells were incubated with 5 *μ*M Akt inhibitor VIII (Santa Cruz Biotechnology sc-203173) for one hour before pretreatment with quercetin and honey extract. Glucose (20 or 50 mM) was added and followed by incubation for another 24 hours. 

### 2.6. Cell Lysate Preparation

After treatment the cells were washed twice in PBS and lysed in mammalian cell lysis buffer (Sigma MCL-1) supplemented with protease and phosphatase inhibitors. Insoluble materials were eliminated by centrifugation (12,000 ×g, 10 min, 4°C), and protein concentration in the supernatant was determined by Bradford assay (Bio-Rad Laboratories).

### 2.7. Measurement of Insulin Content

Insulin content was determined by pretreating 5 × 10^5^ HIT-T15 cells with Gelam honey extract (20, 40, 60, 80 *μ*g/mL) and quercetin (20, 40, 60, 80 *μ*M) for 24 hours following which the medium was replaced with fresh medium. Then glucose of either 20 mM or 50 mM was added and the cells were incubated for another 24 hours. At the end of this incubation, the cells were centrifuged and the supernatant was removed. The cell pellet was resuspended in PBS and sonicated. The cell lysate was used to estimate the quantity of insulin by ELISA (Crystal Chem Inc, USA). Insulin content was normalized to the total protein concentration [29]. 

### 2.8. Western Blot Analysis

Thirty microgram of protein extracts were loaded on 10% SDS-polyacrylamide gel and transferred to activated nitrocellulose membrane. The membrane was blocked with tris-buffered saline (TBS) containing 5% nonfat milk, and incubated with phosphor-Akt (Ser473), phosphor-IRS-1 (Ser307), phospho-p65 NFKB (Ser536), and phospho-p38 MAPK (Thr180/Tyr182) primary antibodies (obtained from Santa Cruz Laboratories) overnight at 4°C. *β*-actin was used as a loading control. After extensive washes in TBS, membranes were incubated for 1 h at room temperature with the appropriate horseradish peroxidase-conjugated secondary antibodies and were visualized using chemiluminescence substrate according to the manufacturer's instructions (Amersham Life Sciences, Little Chalfont, UK). Quantitative analysis of the protein was performed by Gel Documentation System (Biospectrum 410, UVP) [30, 31].

### 2.9. Statistical Analyses

Data were analyzed with one-way ANOVA using SPSS version 16.0 software. The results were expressed as the mean ± standard deviation. *P value < 0.05 was considered to be statistically significant.

## 3. Results

### 3.1. Total Phenolic and Flavonoid Content

10 g of liquefied fresh Malaysian Gelam honey (*Apis mellifera*) was extracted using ethyl acetate and the extract was found to contain 52 *μ*g of gallic acid per gram of extract of total phenolic content and 6.92 *μ*g of quercetin per gram of extract of total flavonoid content.

### 3.2. Effect of Pretreatment with Quercetin and Gelam Honey Extract on Phospho-p38 MAPK Expression under Normal and Hyperglycemic Conditions

**Figure 1:**
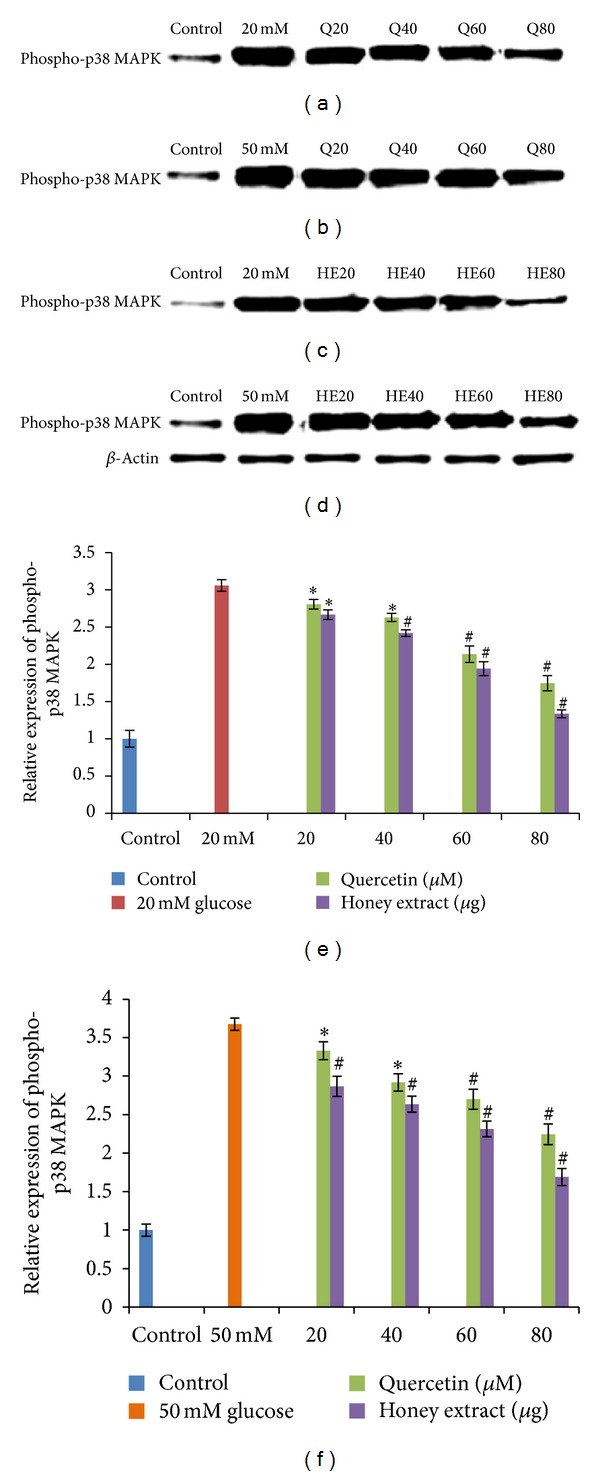
Effect of quercetin and Gelam honey extract on phosphor-p38 MAPK expression. Quantitative analysis and representative western blot analysis of phospho p38 MAPK in HIT-T15 cells pretreated with quercetin and honey extract in cells cultured in 20 mM ((a), (c), (e)) and 50 mM ((b), (d), (f)) glucose. The results were normalized with *β*-actin antibody. Data were presented as the mean ± standard deviation. (e) *P < 0.05; ^#^ *P* < 0.005 quercetin and honey extract treated compared to the 20 mM glucose alone. (f) *P < 0.05; ^#^ *P* < 0.005 quercetin and honey extract treated compared to the 50 mM glucose alone.

HIT-T15 cells were pretreated with the quercetin at concentrations of 20, 40, 60, 80 *μ*M and Gelam honey extract at concentrations of 20, 40, 60, 80 *μ*g/mL for 24 hours, following which they were cultured with 20 mM (Figures 1(a), 1(c), 1(e)) or 50 mM (Figures 1(b), 1(d), 1(f)) glucose to determine the phosphorylation of MAPK. The data revealed that exposure of HIT-T15 cells to 20 and 50 mM glucose significantly increased the level of phospho-p38 MAPK expression compared to control. Pretreatment with quercetin and Gelam honey extract significantly (*P* < 0.05) reduced the ROS-induced expression of phospho-p38 under 20 mM glucose (Figures 1(a), 1(c), 1(e)) by 56% and 40% in a dose-dependent manner. While pretreatment with quercetin and Gelam honey extract reduced the expression of phospho-p38 significantly (*P* < 0.05) by 69% and 44%, respectively, compared to the cells that were cultured with 50 mM glucose (Figures 1(b), 1(d), 1(f)) alone. The p38 MAPK protein levels from each sample were normalized to their respective *β*-actin protein amounts (*P < 0.05; ^#^ *P* < 0.005 versus glucose-treated group).

### 3.3. Effect of Pretreatment with Quercetin and Gelam Honey Extract on Phospho-p65 NF-*κ*B Expression under Normal and Hyperglycemic Conditions

**Figure 2:**
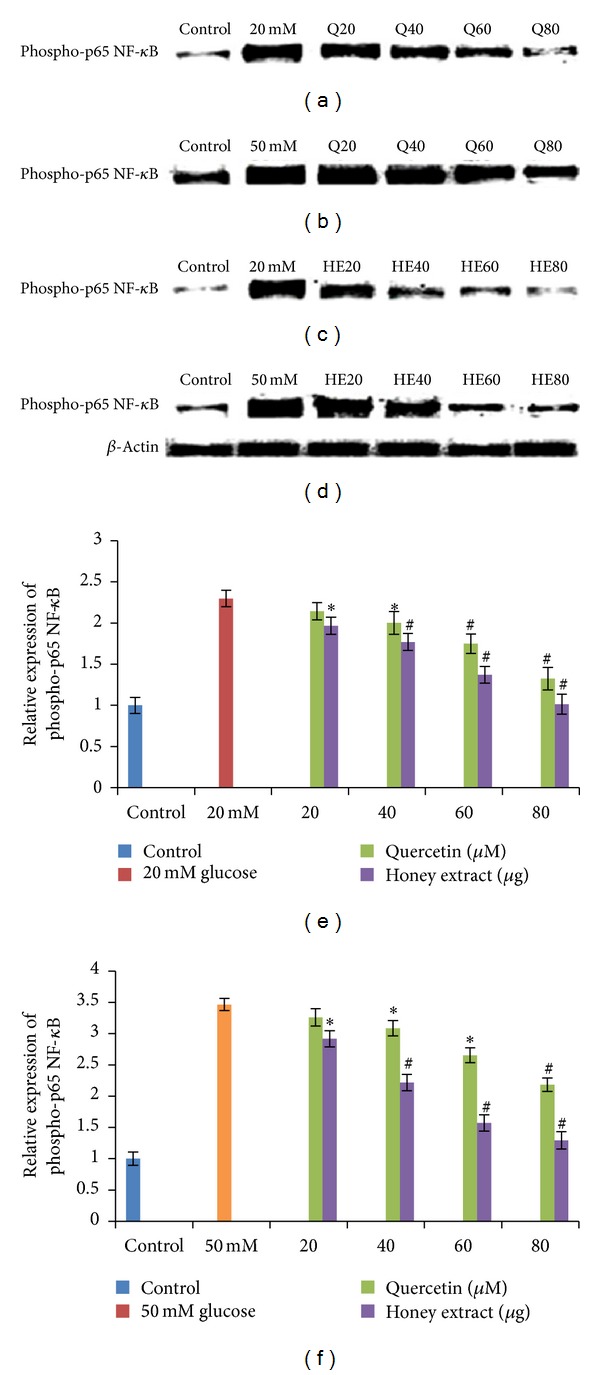
Effect of quercetin and Gelam honey extract on phosphor-p65 NF-*κ*B expression. Quantitative analysis and representative western blot analysis of phospho p65 NF-*κ*B in HIT-T15 cells pretreated with quercetin and honey extract in cells cultured in 20 mM ((a), (c), (e)) and 50 mM ((b), (d), (f)) glucose. The results were normalized with *β*-actin antibody. Data were presented as the mean ± standard deviation. (e) *P < 0.05; ^#^ *P* < 0.005 quercetin and honey extract treated compared to the 20 mM glucose alone. (f) *P < 0.05; ^#^ *P* < 0.005 quercetin and honey extract treated compared to the 50 mM glucose alone.

HIT-T15 cells were pretreated with the quercetin at concentrations of 20, 40, 60, 80 *μ*M and Gelam honey extract at concentrations of 20, 40, 60, 80 *μ*g/mL for 24 hours, following which they were cultured with 20 mM (Figures 2(a), 2(c), 2(e)) or 50 mM (Figures 2(b), 2(d), 2(f)) glucose to determine the phosphorylation of NF-*κ*B. The phosphorylation of NF-*κ*B was increased in HIT-T15 cells treated with 20 mM and 50 mM glucose alone as compared with control. Pretreatment with the quercetin and Gelam honey extract showed a 50% and 56% decrease (*P* < 0.05) in the expression of phosphorylated NF-*κ*B in a dose dependent manner in the cells that were cultured in 20 mM (Figures 2(a), 2(c), 2(e)) glucose. Pretreatment with quercetin and Gelam honey extract significantly (*P* < 0.05) decrease the expression of phosphorylated NF-*κ*B under 50 mM glucose (Figures 2(b), 2(d), 2(f)) by 36% and 61% in a dose dependent manner. The phosphorylated NF-*κ*B protein levels from each sample were normalized to their respective *β*-actin protein amounts (*P < 0.05; ^#^ *P* < 0.005 versus glucose-treated group).

### 3.4. Effect of Pretreatment with Quercetin and Gelam Honey Extract on pIRS-1 (ser307) Expression under Normal and Hyperglycemic Conditions

**Figure 3:**
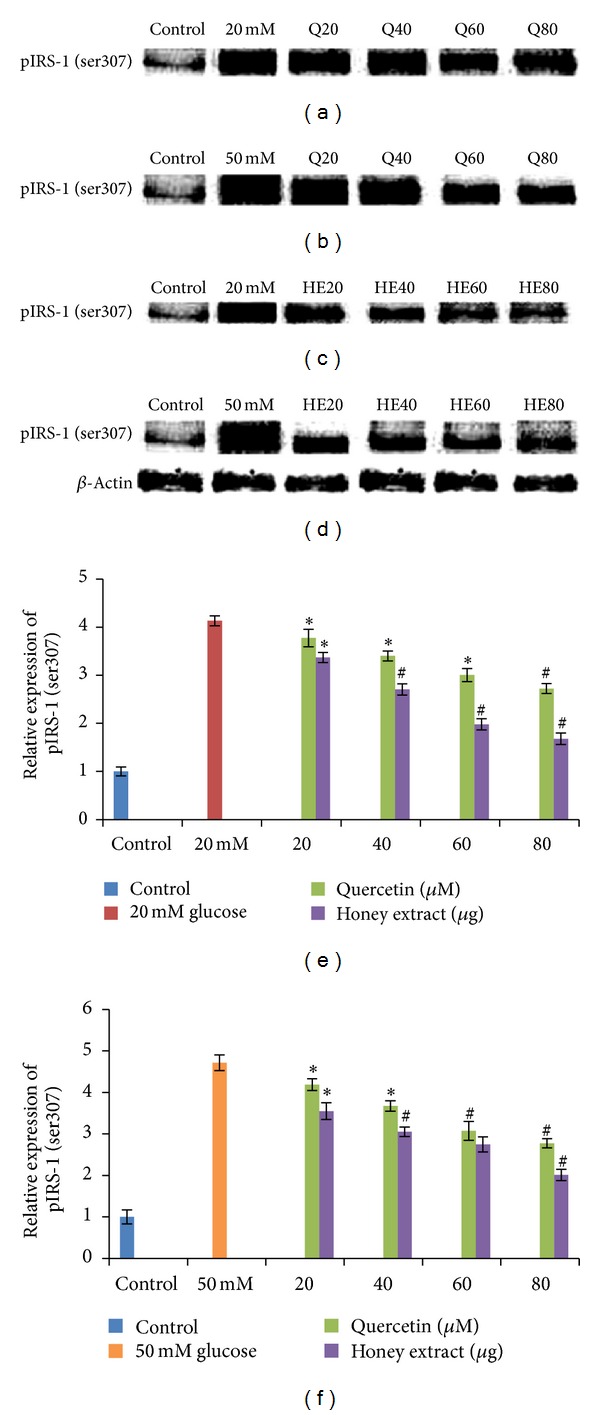
Effect of quercetin on pIRS-1 (ser307) expression. Quantitative analysis and representative western blot analysis of pIRS-1 (ser307) in HIT-T15 cells pretreated with quercetin and Gelam honey extract in cells cultured in 20 mM ((a), (c), (e)) and 50 mM ((b), (d), (f)) glucose. The results were normalized with *β* actin antibody. Data were presented as the mean ± standard deviation. (e) *P < 0.05; ^#^ *P* < 0.005 quercetin and honey extract treated compared to the 20 mM glucose alone. (f) *P < 0.05; ^#^ *P* < 0.005 quercetin and honey extract treated compared to the 50 mM glucose alone.

HIT-T15 cells were pretreated with the quercetin at concentrations of 20, 40, 60, 80 *μ*M and Gelam honey extract at concentrations of 20, 40, 60, 80 *μ*g/mL for 24 hours, following which they were then cultured with 20 mM (Figures 3(a), 3(c), 3(e)) or 50 mM (Figures 3(b), 3(d), 3(f)) glucose to determine the phosphorylation of IRS-1 (ser307). The phosphorylation of IRS-1 (ser307) was increased in HIT-T15 cells treated with 20 mM and 50 mM glucose alone as compared with control. Pretreatment with the quercetin and Gelam honey extract showed a 46% and 52% decrease (*P* < 0.05) in the expression of pIRS-1 (ser307) in a dose-dependent manner in the cells that were cultured in 20 mM glucose (Figures 3(a), 3(c), 3(e)). Pretreatment with quercetin and Gelam honey extract significantly (*P* < 0.05) decreased the expression of pIRS-1 (ser307) under 50 mM glucose (Figure 3(b), 3(d), 3(f)) by 40% and 50% in a dose dependent manner. The pIRS-1 protein levels from each sample were normalized to their respective *β*-actin protein amounts (*P < 0.05; ^#^ *P* < 0.005 versus glucose-treated group).

### 3.5. Effect of Pretreatment with Quercetin and Gelam Honey Extract on pAkt (ser473) Expression under Normal and Hyperglycemic Conditions

**Figure 4:**
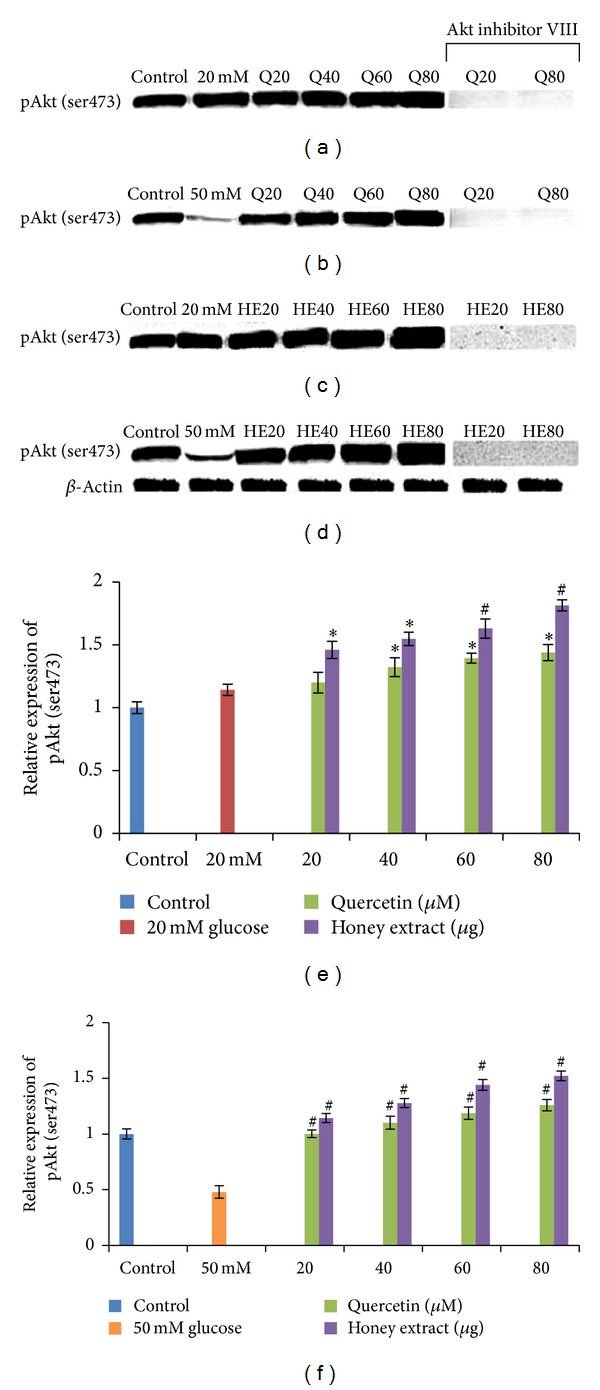
Effect of quercetin and Gelam honey extract on pAkt (Ser473) expression.Quantitative analysis and representative western blot analysis of pAkt (Ser473) in HIT-T15 cells pretreated with quercetin and honey extract in cells cultured in 20 mM ((a), (c), (e)) and 50 mM ((b), (d), (f)) glucose. A sustained increase in the level of pAkt (ser473) was observed after pretreatment with quercetin and honey extract. Akt inhibitor VIII prevented the expression Akt ser473 phosphorylation induced by quercetin and honey extract. The results were normalized with *β* actin antibody. Data were presented as the mean ± standard deviation. (e) *P < 0.05; ^#^ *P* < 0.005 quercetin and honey extract treated compared to the 20 mM glucose alone. (f) *P < 0.05; ^#^ *P* < 0.005 quercetin and honey extract treated compared to the 50 mM glucose alone.

HIT-T15 cells were pretreated with the quercetin at concentrations of 20, 40, 60, 80 *μ*M and Gelam honey extract at concentrations of 20, 40, 60, 80 *μ*g/mL for 24 hours, following which they were then cultured with 20 or 50 mM glucose to determine the phosphorylation of Akt (ser473). Akt (ser473) phosphorylation in HIT-T15 cells was markedly reduced following 20 mM (Figures 4(a), 4(c), 4(e)) and 50 mM (Figures 4(b), 4(d), 4(f)) glucose treatment, but the trend was reversed after pretreatment with quercetin and Gelam honey extract. Pretreatment of the cells with different concentration of quercetin and honey extract for 24 hours significantly increase the expression of pAkt (ser473) up to 32% and 70% respectively, compared to the cells that were cultured alone with 20 mM glucose (Figure 4(a), 4(c), 4(e)). On the other hand, pretreatment with quercetin and honey extract increases the expression of pAkt (ser473) significantly (*P* < 0.05) up to 19% and 54%, respectively, compared to the cells that were cultured alone with 50 mM glucose (Figures 4(b), 4(d), 4(f)). The cells that were exposed to Akt inhibitor VIII prevented the quercetin and honey extract-induced Akt ser473 phosphorylation. The pAkt protein levels from each sample were normalized to their respective *β*-actin protein amounts (*P < 0.05; ^#^ *P* < 0.005 versus glucose-treated group). 

### 3.6. The Effect of Quercetin and Gelam Honey Extract and Akt Inhibitor on Insulin Content of the Cells

**Figure 5:**
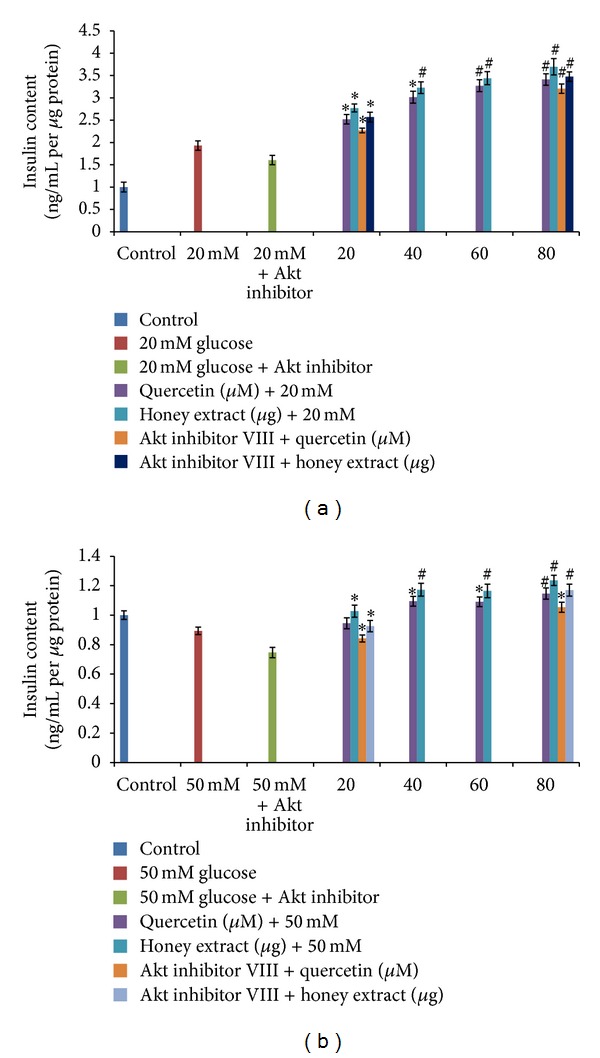
The effect of flavonoids and Gelam honey extract on insulin content. (a) Effect of pretreatment with quercetin and Gelam honey extract and the addition of Akt inhibitor VIII on the insulin content in cells cultured in 20 mM glucose. There was a significant increase in insulin content (*P < 0.05) when the cells were pretreated with quercetin and honey. There was a significant decrease in insulin content (*P < 0.05) when the cells were treated with Akt inhibitor VIII, before pretreating with quercetin and Gelam honey extract. (b) Effect of pretreatment with quercetin and Gelam honey and the addition of Akt inhibitor VIII on the insulin content in cells cultured in 50 mM glucose. There was a significant increase in insulin content (*P < 0.05, ^#^ *P* < 0.005) when the cells were pretreated with quercetin and honey. There was a significant decrease in insulin content (*P < 0.05, ^#^ *P* < 0.005) when the cells were treated with Akt inhibitor VIII, before pretreating with quercetin and Gelam honey extract.

To determine the effect of ROS on insulin content, the cells were pretreated with Gelam honey extract (20, 40, 60, 80 *μ*g/mL) and the quercetin (20, 40, 60, 80 *μ*M) before treating the HIT-T15 cells with 20 mM and 50 mM glucose and the insulin content was measured. As shown in Figure 5(a), pretreatment with quercetin and Gelam honey extract increased the insulin content significantly (*P* < 0.05) up to 64% and 78%, respectively, compared to the cells that were cultured alone with 20 mM glucose.  Figure 5(b) shows that pretreatment with quercetin and Gelam honey extract increased the insulin content significantly (*P* < 0.05) up to 34% and 48% respectively, compared to the cells that were cultured alone with 50 mM glucose. Exposure of cells to Akt inhibitor VIII for 1 hour before pretreatment with quercetin (20, 80 *μ*M) and Gelam honey extract (20, 80 *μ*g/mL) decreases the insulin content significantly (*P* < 0.05) up to 12% and 6% respectively, compared to the cells that were pretreated with quercetin and honey extract cultured in 20 mM glucose (Figure 5(a)) without Akt inhibitor. The cells that were exposed to Akt inhibitor VIII for 1 hour and pretreated with quercetin (20, 80*μ*M) and Gelam honey extract (20, 80 *μ*g/mL) decrease the insulin content significantly (*P* < 0.05) up to 10% and 8% respectively, compared to the cells that were pretreated with quercetin and honey extract before culturing in 50 mM glucose (Figure 5(b)) without Akt inhibitor.

## 4. Discussions

In our previous study, we investigated the antioxidant effect of the Malaysian Gelam honey and some of its flavonoid components (chrysin, luteolin, and quercetin) individually on pancreatic hamster cells (HIT-T15) cultured under hyperglycemic conditions. Our data demonstrated that the cultured cells, pretreated with the extract of the Gelam honey and the different flavonoid components (quercetin, luteolin, and chrysin) at varying concentrations for 24 hours, protected the *β* cell from oxidative damage caused by ROS induced by hyperglycemia [24]. Therefore in our present study, we determined the effect of Gelam honey extract and quercetin on the stress-activated NF-*κ*B, MAPK pathways and IRS-1 serine phosphorylation causing insulin resistance and the Akt-activated insulin signaling pathway, causing increase in insulin content. 

Several studies have demonstrated that flavonoids may reduce hyperglycemia and exert protective effects against nonenzymatic glycation of proteins in animals [32, 33]. Two important studies in streptozotocin-(STZ-) induced diabetes mellitus [33] and alloxan-induced diabetes mellitus in rats [25] have demonstrated that quercetin may even reverse the hyperglycemia close to the normal levels. More recently hyperglycemia has been implicated in the stress-activated signaling pathways such as p-65 NF-*κ*B and p-38 MAPK [17]. Activation of these pathways is linked not only to the development of the late complications of diabetes, but also to insulin resistance and *β*-cell dysfunction [16]. 

Another study on bovine endothelial cells has revealed that exposure to hyperglycemia initially increased the production of intracellular ROS, followed by activation of p-65 NF-*κ*B, subsequently increasing the PKC activity, the advanced glycation end products (AGE), and sorbitol levels. It has been shown that when hyperglycemia-induced ROS production was reduced, the hyperglycemia-induced effects on NF-*κ*B, PKC, AGE, and sorbitol were also suppressed [34, 35]. In addition, *α*-phenyl-tert-butylnitrone, a spin-trapping agent that reacts with ROS, significantly reduces the severity of hyperglycemia in both alloxan- and streptozotocin-induced diabetes and inhibits the activation of p-65 NF-*κ*B [36]. It has been shown that both the activation of p-65 NF-*κ*B and the increase in oxidative stress are reduced in rats fed on a diet supplemented with multiple antioxidants [37]. These data indicate that activation of p-65 NF-*κ*B is an initial signaling event caused by ROS that leads to cellular dysfunction and damage [36]. Our findings suggest that in pancreatic hamster cell, quercetin and Gelam honey extracts are able to reduce p-38 MAPK and p-65 NF-*κ*B activation, by its antioxidant effect on ROS. This gives further support that oxidative stress is the initial change induced by high glucose. Our data shows that exposure of HIT-T15 cells to 20 and 50 mM glucose caused a significant increase level of phospho-p38 MAPK (Figure 1) expression and phospho-p65 NF-*κ*B (Figure 2) expression compared to control. Pretreatment with quercetin and Gelam honey extract significantly (*P* < 0.05) reduced the ROS-induced expression of phospho-p38 MAPK (Figure 1) and phospho-p65 NF-*κ*B (Figure 2) under 20 and 50 mM glucose.

Activation of p38 MAPK has been suggested as one of the potential candidates for mediating IRS-1 serine phosphorylation (ser307) by cellular stresses [38–40] causing steric hindrance of the interaction between IR and IRS-1. Our data shows that phosphorylation of IRS-1 (ser307) was increased in HIT-T15 cells treated with 20 and 50 mM glucose alone as compared with control. Pretreatment with the quercetin and Gelam honey extract significantly decreased (*P* < 0.05) the expression of pIRS-1 (ser307) (Figure 3) in a dose-dependent manner in the cells that were cultured in 20 and 50 mM glucose (Figure 3). There is possibility that ROS generation, in response to stress stimuli, may promote IRS-1 (ser307) phosphorylation.

Cho et al. in 2001 reported that mice lacking the p-Akt (ser473) protein are insulin resistant, with impaired insulin secretion [41]. Ernesto et al. reported that Akt (ser473) is necessary for normal pancreatic *β*-cell function and described a novel regulatory role for Akt signaling in insulin secretion [42]. Several studies reported that a decrease in insulin secretion and insulin resistance induced by hyperglycemia has been associated with decreased Akt activity [19, 20, 41, 43, 44]. Our data validates the above statement by showing that Akt (ser 473) (Figure 4) phosphorylation in HIT-T15 cells was markedly reduced following 20 and 50 mM glucose treatment but was reversed after pretreatment with quercetin and Gelam honey extract. Our data suggest that Akt (ser473) (Figure 4) phosphorylation and insulin content (Figure 5) were increased after pre-treatment with quercetin and honey showing the protective effects against *β* cell dysfunction. The present data shows that treatment with an Akt inhibitor decreased the insulin content significantly (Figure 5) supporting the previous data reported by Cordero et al. [45] that Cocoa flavonoids improve insulin signaling and modulate glucose production via Akt in HepG2 cells.

 Growing evidence indicates that ROS are involved in maintaining normal *β*-cell glucose responsiveness. ROS may have different actions depending on whether the cellular concentrations are either below or above a specific threshold, that is, signaling versus toxic effects. H_2_O_2_ derived from glucose metabolism is one of the metabolic signals for insulin secretion. Also it has been shown that mitochondrial reactive oxygen species are obligatory signals for glucose-induced insulin secretion. ROS produced under short-term exposure, or under nonhyperglycemic conditions may play a role in physiological regulation of glucose induced insulin secretion, while long-term exposure to high glucose induces oxidative stress in *β* cells [24]. Our data validate the above statement by showing that cells grown under 20 mM glucose (Figure 5(a)) under nonhyperglycemic conditions showed an increase in insulin content compared to the controls, while in cells grown under 50 mM glucose (Figure 5(b)) concentration, the insulin content was reduced significantly as compared to controls.

 In conclusion, our data suggest the potential use of the extract from Gelam honey in treating diabetes, by modulating the oxidative stress-induced insulin signalling pathways. The data provide further support for the implication of oxidative stress, in *β*-cell dysfunction. Further studies are required to accurately define the mechanisms involved in diabetic complications at the molecular and biochemical levels.
